# Magneto-transport properties of a single molecular transistor in the presence of electron-electron and electron-phonon interactions and quantum dissipation

**DOI:** 10.1038/s41598-019-53008-5

**Published:** 2019-11-11

**Authors:** Manasa Kalla, Narasimha Raju Chebrolu, Ashok Chatterjee

**Affiliations:** 1 School of Physics, University of Hyderabad, Hyderabad, 500046 India; 2 Physics Department, University of Seoul, Seoul, South Korea

**Keywords:** Electronic properties and materials, Molecular electronics

## Abstract

A single molecular transistor is considered in the presence of electron-electron interaction, electron-phonon interaction, an external magnetic field and dissipation. The quantum transport properties of the system are investigated using the Anderson-Holstein Hamiltonian together with the Caldeira-Leggett model that takes care of the damping effect. The phonons are first removed from the theory by averaging the Hamiltonian with respect to a coherent phonon state and the resultant electronic Hamiltonian is finally solved with the help of the Green function technique due to Keldysh. The spectral function, spin-polarized current densities, differential conductance and spin polarization current are determined.

## Introduction

Lately a surge of activity has been witnessed on single molecular transistors^1–4^ for they can play an important role in nano-electronics. A single molecular transistor (SMT) is a nano-device that contains at the centre a molecule or a quantum dot (QD) characterized by discrete energy levels and coupled to a source and a drain by metallic leads. The current through an SMT device can be effectively controlled by tuning the gate voltage^5–7^. The first SMT device was fabricated in 2000 by using a single C_60_ molecule^8,9^ bridging the source and the drain. Many research groups have found that the transport through SMT exhibits at low temperature correlation effects like Coulomb blockade and Kondo effect^10–16^. In polar QDs, however, the interaction of electrons with phonons gives rise to polarons which are electrons dressed with phonons and therefore the quasi-particles that take part in transport mechanism in these systems are polarons^17–24^. Thus the transport properties of an SMT device are affected in general by both electron-electron (e-e) and electron-phonon (e-p) interactions. Chen *et al*.^25^ have observed that e-p interaction produces side bands in the spectral function and makes the zero-phonon peak sharper. They have also analyzed how the quantum transport properties like tunneling current and differential conductance would depend on the chemical potentials of the leads at zero temperature.

The quantum transport properties of SMT have been studied by using different theoretical and numerical methods like kinetic equation method^26,27^, rate equation approach^28^, slave-boson mean-field method^29^, non-crossing approximation method^30^, numerical renormalization method^31–35^ and non-equilibrium Green’s function approaches^36–40^. Raju and Chatterjee have investigated in a recent work^41^, the effect of dissipation on quantum transport in an SMT device employing the Keldysh non-equilibrium Green function formalism at zero temperature. To describe the SMT system mounted on a substrate they have considered the Anderson-Holstein (AH) Hamiltonian together with the Caldeira-Leggett (CL) term (hereafter referred to as AHCL model) which introduces a linear dissipative coupling between the phonons of the substrate and that of the QD. Their calculation suggests that the SMT parameters are renormalized by the polaronic effects induced by the e-p interaction. This leads to a shift of the spectral function and differential conduction peaks. Also the peaks become sharper. Furthermore, the e-p coupling reduces the tunneling current, while the phonon dissipation enhances it. Costi^42^ has shown with the help of numerical renormalization group technique of Wilson, that the transport properties of electrons through the QD of an SMT device can be modified by a magnetic field leading to visible effects in the transport properties. It has been further suggested that a strongly coupled QD in an external field can act as a spin filter whose properties can be controlled by tuning the gate voltage. Dong *et al*.^43^ have found that an externally applied magnetic field suppresses the zero-temperature linear conductance. They have also observed that the magnetic field induces side peaks in the conductance if it is increased sufficiently.

In this work we purport to investigate the magnetic field effect on the quantum transport properties of an SMT by employing the Keldysh Green function formalism. We employ the AHCL Hamiltonian to model the system and calculate the tunneling current. We also explore how the other SMT properties like spectral density, spin-polarized differential conductance and spin polarization current are influenced by an externally applied magnetic field.

## The Model

**Figure 1:**
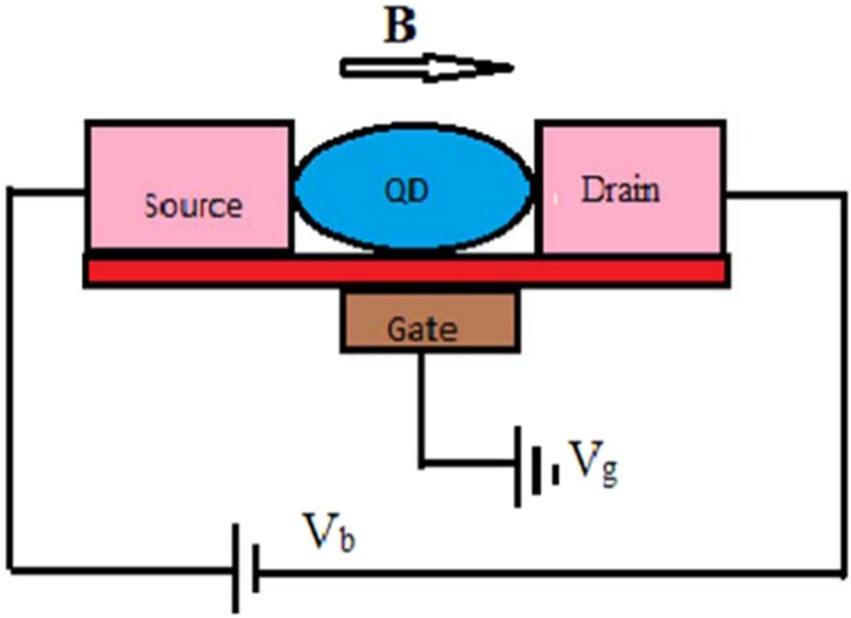
Schematic diagram of an SMT device.

As has been already mentioned, an SMT system contains a QD connected to a source (S) and a drain (D) by two metallic leads. We assume that QD has a single lattice mode interacting with the electrons through the e-p coupling of Holstein type. The structure consisting of the QD together with the source and drain system is mounted on a substrate which is an insulator and acts as a heat-bath. Thus the phonons of the substrate interact with the QD phonon mode through a linear CL interaction which gives rise to a dissipative effect to the current in SMT. Figure 1 represents a schematic diagram of an SMT system in an externally applied magnetic field. The transport properties of the QD are expected to get modified by the applied magnetic field and such effects have indeed become visible^44,45^. Due to the lifting of spin degeneracy of the QD by the external field, the QD setup can act as a spin filtering device and produce a current that is spin-polarized. The system can be modeled by the Hamiltonian

1 $$H={H}_{l}+{H}_{QD}+{H}_{t}+{H}_{B}.$$

In Eq. (1), the Hamiltonian *H*_*l*_ describes the source (*l* = S) and the drain (*l* = D):

2 $${H}_{l}=\sum _{k\sigma \in S,D}\,{\varepsilon }_{k}{n}_{k\sigma },$$

where ${n}_{k\sigma }(\,={c}_{k\sigma }^{\dagger }{c}_{k\sigma })\,\,$represents the number operator for the free electrons in the momentum state ***k*** and spin *σ* in the metallic leads, ${c}_{k\sigma }^{\dagger }({c}_{k\sigma })$ being the corresponding electron creation (annihilation) operator. *H*_*QD*_ refers to the QD Hamiltonian given by

3 $${H}_{{\rm{QD}}}=\sum _{\sigma }\,({\varepsilon }_{d}-e{V}_{g}){n}_{d\sigma }+U{n}_{d,\sigma }{n}_{d,-\sigma }+\frac{1}{2}g{\mu }_{B}B{S}_{d}^{z}+\hslash {\omega }_{0}{b}^{\dagger }b+\,\lambda \hslash {\omega }_{0}({b}^{\dagger }+b)\,\sum _{\sigma }\,{n}_{d\sigma },$$

where ${n}_{d\sigma }(\,=\,{c}_{d\sigma }^{\dagger }{c}_{d\sigma })$ represents the number operator for the QD electrons with energy *ε*_*d*_, ${c}_{d\sigma }^{\dagger }({c}_{d\sigma })$ being the corresponding electron creation (annihilation) operator, *V*_*g*_ denotes the gate voltage, *U* gives the measure of the onsite e-e interaction, ***B*** (0, 0, *B*) refers to the magnetic field, ${S}_{d}^{z}$ stands for the total spin of the QD electrons along the z direction, *b*^†^(*b*) creates (destroys) a QD phonon with frequency *ω*_0_ which is considered dispersionless and *λ* denotes the e-p coupling constant. The Hamiltonian *H*_*t*_ describes the hybridization of the QD electrons and the leads electrons and is given by

4 $${H}_{t}=\sum _{k\sigma \epsilon S,D}\,({V}_{k}{c}_{k\sigma }^{\dagger }{c}_{d\sigma }+h.\,c),$$

where *V*_*k*_ is the hybridization coefficient which essentially determines the strength of electron tunneling between the QD and the source or drain. The SMT system is mounted on an insulating substrate which is considered as a phonon bath and can thus be expressed as a collection of uncoupled harmonic oscillators. The phonon bath and its interaction with the local QD phonon can be written following the CL model as

5 $${H}_{B}=\mathop{\sum }\limits_{j=1}^{N}\,[\frac{{p}_{j}^{2}}{2{m}_{j}}+\frac{1}{2}{m}_{j}{\omega }_{j}^{2}{x}_{j}^{2\,}]+\mathop{\sum }\limits_{j=1}^{N}\,{\beta }_{j}{x}_{j}{x}_{0},$$

where *x*_*j*’s_ and *x*_0_are the generalized coordinates of the substrate oscillators and the QD respectively, *ω*_*j*_ represents the frequency of the *j*-th oscillator of the substrate and *β*_*j*_ denotes the strength of the coupling between the *j*-th oscillator of the substrate and the oscillator of QD. The spectral function of the phonons of the substrate (*J*(*ω*)) is described by the spectral function: $J(\omega )=\mathop{\sum }\limits_{j=1}^{N}\,[{\beta }_{j}^{2}/(2{m}_{j}{\omega }_{j})]\delta (\omega -{\omega }_{j}).$

## Formulation

First of all, the linear coupling between local the QD phonon and the bath phonons is removed by performing a unitary transformation. As a result, the frequency of the local phonon frequency gets renormalized to ${\tilde{\omega }}_{0}=\sqrt{({\omega }_{0}^{2}-\Delta {\omega }^{2})},$ where Δ*ω*^2^ = 2*πγω*_*c*_, *ω*_*c*_ defining the cut-off frequency and *γ* the dissipation rate. The total transformed Hamiltonian reads as

6 $$\begin{array}{c}\bar{H}=\sum _{k\sigma \epsilon S,D}\,{\varepsilon }_{k}{n}_{k\sigma }+\sum _{\sigma }\,({\varepsilon }_{d}-e{V}_{g}){n}_{d\sigma }+U{n}_{d,\sigma }{n}_{d,-\sigma }+g{\mu }_{B}B{S}_{d}^{z}\\ \,\,\,\,\,+\,\hslash {\tilde{\omega }}_{0}{b}^{\dagger }b+\lambda \hslash {\tilde{\omega }}_{0}({b}^{\dagger }+b)\sum _{\sigma }\,{n}_{d\sigma }+\sum _{k\sigma \epsilon S,D}\,({V}_{k}{c}_{k\sigma }^{\dagger }{c}_{d\sigma }+h.\,c).\end{array}$$

To eliminate the e-p coupling, we next apply to Eq. (6) the celebrated Lang-Firsov transformation (LFT)^46^ *e*^*S*^, where $S=\lambda ({b}^{\dagger }-b)\,\sum _{\sigma }\,{n}_{d\sigma }$. It is well-known that this transformation works better for the anti-adiabatic regime. The effective Hamiltonian becomes

7 $$\tilde{H}=\sum _{k\sigma \epsilon S,D}\,{\varepsilon }_{k}{n}_{k\sigma }+\sum _{\sigma }\,{\tilde{\varepsilon }}_{d}{n}_{d\sigma }+\tilde{U}{n}_{d,\sigma }{n}_{d,-\sigma }+\hslash {\tilde{\omega }}_{0}{b}^{\dagger }b+\sum _{k\sigma \epsilon S,D}\,({\tilde{V}}_{k}{c}_{k\sigma }^{\dagger }{c}_{d\sigma }+h.\,c),$$

with

8 $${\tilde{\varepsilon }}_{d\sigma }={\varepsilon }_{d}-e{V}_{g}-{\mu }_{B}\sigma B-{\lambda }^{2}\hslash {\tilde{\omega }}_{0},\,\tilde{U}=U-2{\lambda }^{2}\hslash {\tilde{\omega }}_{0},\,{\tilde{V}}_{k}={V}_{k}{e}^{\lambda ({b}^{\dagger }-b)},$$

where ${\tilde{\varepsilon }}_{d\sigma }$ is the QD energy renormalized by the e-p interaction, $\tilde{U}$ denotes the modified Coulomb correlation strength and ${\tilde{V}}_{k}$ represents the phonon-mediated hybridization strength. The tunneling current^36,47^ flowing through the central interacting QD is obtained as

9 $$J=\frac{e}{2h}\int \{{f}_{s}{\Gamma }_{s}-{f}_{D}{\Gamma }_{D}\}A(\varepsilon )+({\Gamma }_{S}-{\Gamma }_{D}){G}^{ < }(\varepsilon )d\varepsilon .$$

Here ${\Gamma }_{S,D}({\varepsilon }_{k})=2\pi {\varrho }_{S,D}({\varepsilon }_{k}){\tilde{V}}_{k}{V}_{k}^{\ast },$_*ρS*(*D*)_ defining the density of energy states in the source (drain), *f*_*S*(*D*)_*(ε*) denotes the Fermi function of the source (drain), the corresponding chemical potentials of which are connected to the bias voltage (*V*_*B*_) and mid-voltage (*V*_*m*_) by the equations: *(μ*_*S*_ −* μ*_*D*_) = *eV*_*B*_, *(μ*_*S*_ + *μ*_*D*_)/2 = *eV*_*m*_, A(*ε*) is the spectral function which describes the excitations and is related to the Green functions as

10 $${\rm{A}}(\varepsilon )=i[{G}_{dd}^{r}(\varepsilon )-{G}_{dd}^{a}(\varepsilon )]=i[{G}_{dd}^{ < }(\varepsilon )-{G}_{dd}^{ > }(\varepsilon )],$$

where ${G}_{dd}^{r(a)}(\varepsilon )$ represents the retarded (advanced) Green function and $\,{G}_{dd}^{ < ( > )}(\varepsilon )$ refers to the lesser (greater) Keldysh Green function for the QD electron in the energy space. They are the Fourier transforms of ${G}_{dd}^{r(a)}(\tau =t-t^{\prime} )$ and ${G}_{dd}^{ < ( > )}(\tau =t-t^{\prime} )$ which are defined as

11 $$\begin{array}{c}{G}_{dd}^{r(a)}(t-t^{\prime} )=\mp \,i\,\theta (\,\pm \,t\mp t^{\prime} )\langle 0|\{{\tilde{c}}_{d}(t),{\tilde{c}}_{d}^{\dagger }(t^{\prime} )\}|0\rangle ,\\ {G}_{dd}^{ < }(t-t^{\prime} )=i\langle 0|{c}_{d}^{\dagger }(t^{\prime} ){c}_{d}(t)|0\rangle ,\,{G}_{dd}^{ > }(t-t^{\prime} )=i\langle 0|{c}_{d}(t)\,{c}_{d}^{\dagger }(t^{\prime} )|0\rangle ,\end{array}$$

with ${c}_{d\sigma }(t)={e}^{-i{\tilde{H}}_{el}t}{c}_{d\sigma }{e}^{i{\tilde{H}}_{el}t}$, ${\tilde{c}}_{d\sigma }(t)=\hat{O}{c}_{d\sigma }$, where $\hat{O}={e}^{-\lambda ({b}^{\dagger }-b)}$ and |0〉 stands for the exact ground state of the whole system i.e., |0〉 = |0〉_*el*_|0〉_*ph*_. The average occupancy on the QD can be calculated using the relation

12 $${n}_{d\sigma }=-\,{Im}\,{G}_{dd}^{ < }(\tau )=\frac{1}{2\pi }\int d\varepsilon \frac{\{{f}_{s}{\Gamma }_{s}+{f}_{D}{\Gamma }_{D}\}}{\Gamma }{\rm{A}}(\varepsilon ).$$

We assume that the QD is symmetrically coupled to the left and the right leads. Therefore we can write: Γ(*ε*) = (Γ_*S*_(*ε*) + Γ_*D*_(*ε*))/2, where we write for Γ_*S*(*D*)_ its average value which is given by expression: ${\Gamma }_{S(D)}=2\pi \rho (0){|{V}_{k}|}^{2}{e}^{-{\lambda }^{2}/2}$. The spectral density function is determined by considering one particle Green functions. One can show^47^

13a $$\begin{array}{c}{G}_{dd}^{ < }(\tau )=i\langle 0|{c}_{d}^{\dagger }(0){\mathop{c}\limits^{ \sim }}_{d}(\tau )|0\rangle =i\langle 0|{c}_{d}^{\dagger }(0){c}_{d}(\tau ){|0\rangle }_{el}{\langle {\hat{O}}^{\dagger }\hat{O}\rangle }_{ph}\\ \,\,\,\,\,\,={\mathop{G}\limits^{ \sim }}_{dd}^{ < }(\tau ){e}^{-\phi (-\tau )}={\mathop{G}\limits^{ \sim }}_{dd}^{ < }(\tau )\mathop{\sum }\limits_{n=-{\rm{\infty }}}^{{\rm{\infty }}}\,{L}_{n}{e}^{in\hslash {\mathop{\omega }\limits^{ \sim }}_{0}\tau },\end{array}$$

13b $${G}_{dd}^{ > }(\tau )=-\,i\langle 0|{\mathop{c}\limits^{ \sim }}_{d}(0){\mathop{c}\limits^{ \sim }}_{d}^{\dagger }(\tau )|0\rangle =-\,i\langle 0|{c}_{d}(0){c}_{d}^{\dagger }(\tau ){|0\rangle }_{el}{\langle \hat{O}{\hat{O}}^{\dagger }\rangle }_{ph}={\mathop{G}\limits^{ \sim }}_{dd}^{ > }(\tau )\mathop{\sum }\limits_{n=-{\rm{\infty }}}^{{\rm{\infty }}}\,{L}_{n}{e}^{in\hslash {\mathop{\omega }\limits^{ \sim }}_{0}\tau },$$

where

14 $$\phi (\,\mp \,\tau )={\lambda }^{2}[(2{f}_{ph}+1)\mp {({f}_{ph}(1+{f}_{ph}))}^{1/2}2\,\cos \,(\hslash {\tilde{\omega }}_{0}(\,\mp \,\tau +i\beta /2))],$$

*f*_*ph*_ being the phonon distribution function and at zero temperature: ${L}_{n}={\lambda }^{2n}/n!\,{e}^{-{\lambda }^{2}}$ for *n* ≥ 0 and *L*_*n*_ = 0 for *n* < 0. Taking the Fourier transform of Eqs (13a) and (13b), we can write the spectral density function of the SMT device as

15 $${\rm{A}}(\varepsilon )=\mathop{\sum }\limits_{n=-\infty }^{\infty }\,i{L}_{n}(z)[{\tilde{G}}^{ > }(\varepsilon -n\hslash {\tilde{\omega }}_{0})-{\tilde{G}}^{ < }(\varepsilon +n\hslash {\tilde{\omega }}_{0})].$$

where *n* denotes the number of phonons. Using the analytical continuation rules of Langreth to the Dyson equations for the Green functions ${G}_{dd}^{ < ( > )}(\varepsilon )$, one can write

16 $${\tilde{G}}^{ > ( < )}(\varepsilon )={\tilde{G}}_{dd}^{r}(\varepsilon )\,{\Sigma }^{ > ( < )}(\varepsilon ){\tilde{G}}_{dd}^{a}(\varepsilon ),$$

where

17 $${\Sigma }^{ < }(\varepsilon )=i\,\Gamma [{f}_{S}(\varepsilon )+{f}_{D}(\varepsilon )],\,{\Sigma }^{ > }(\varepsilon )=-\,i\,\Gamma [2-({f}_{S}(\varepsilon )+{f}_{D}(\varepsilon ))].$$

The Green functions ${\tilde{G}}_{dd}^{r,a}(\varepsilon )$ can be calculated exploiting the equation of motion technique^48,49^. We apply a mean-field approximation (MFA) within the framework of Hartree-Fock (HF) decoupling scheme to treat the on-site Hubbard Coulomb-correlation term. Thus our results would be well outside the Kondo regime. Then ${\tilde{G}}^{ > ( < )}(\varepsilon )$ and A(*ε*) can be calculated and hence the tunneling current can be determined. We also calculate the differential conductance (*G* = *dJ*/*dV*_*b*_)and spin polarization parameter: *P*_*σ*, −*σ*_ = (*J*_*σ*_ − *J*_−*σ*_)/(*J*_*σ*_ + *J*_−*σ*_).

## Results and Discussions

**Figure 2:**
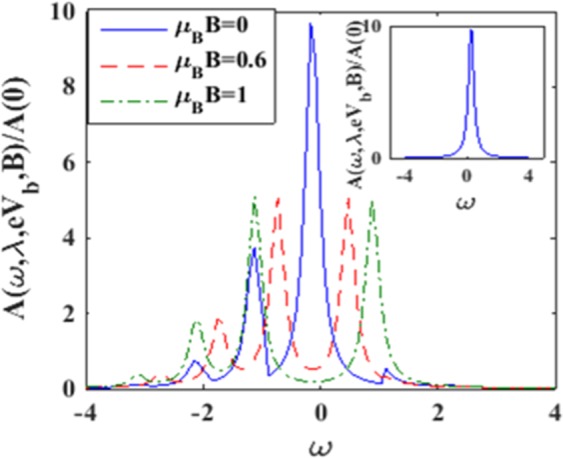
A (*ω*, *λ*, e*V*_*b*_, *B*)/A(0) vs *ω* for *γ* = 0.02, *eV*_*b*_ = 0.5, *λ* = 0.6 for different values of *μ*_*B*_*B*. (Inset: A (*ω*, *λ*, *eV*_*b*_, *B*)/A(0) for *λ* = 0 = *γ*, *eV*_*b*_ = 0.5 and *B* = 0).

For simplicity, we assume QD to contain a single level with energy *ε*_*d*_ = 0 and is connected symmetrically to the source and the drain. From now on, we choose the phonon energy *ħω*_0_ as the unit of energy and set Γ = 0.2, *eV*_*g*_ = 0, *k*_*B*_*T* = 0, *ħω*_0_ = 1. Also we choose *U* = 5 for most parts of the computations. Though *U* = 5 may seem to be a little large, but since after the application of LFT, the on-site Coulomb correlation strength gets renormalized to $\tilde{U}$ which may be quite small (because of the polaronic effect), the HF MFA can be considered to be a good enough approximation for the present problem. Also we assume that the energy density of states for the electrons of the source and the drain that participate in the conduction process can be considered to be constant. Figure 2 displays behaviour of the spectral density *A* in the presence of e-e interaction, e-p interaction and dissipation for a few values of the magnetic field strength *B*. The inset shows the behavior of the same function *A* for *λ* = 0 = *γ* in the case of *U* = 0, and *B* = 0^41^. The behavior is Lorenzian with a single resonance peak at *ε*_*d*_ = 0. Raju and Chatterjee^41^ have studied the spectral function for non-zero values of *λ* and *γ* with *B* = 0. Their results are also plotted in Figure 2 which shows a central peak and a few side bands due to polaronic effects as expected. In the case of *B* ≠ 0, the central peak is split and side peaks shift to the left. It is well known that a peak appearing in the spectral function implies the possibility of an excitation. In *B* = 0 case, the states corresponding to spin-up and spin-down electrons have the same energy and the externally applied magnetic field lifts this spin-degeneracy giving rise to the splitting of the central peak of the spectral function.

**Figure 3:**
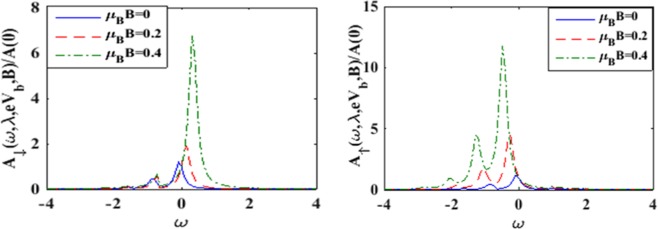
*A*_↓_ and *A*_↑_ *vs ω* for different values of *B* at *γ* = 0.02, *eV*_*b*_ = 0.5 *and λ* = 0.6.

**Figure 4:**
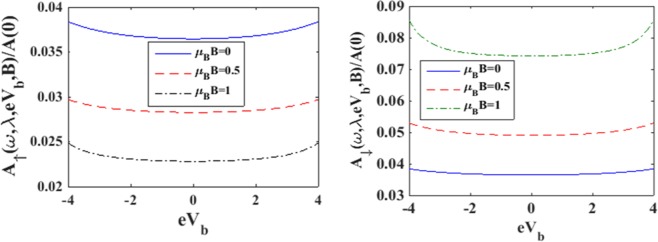
*A*_↑_ *and A*_↓_ *vs eV*_*b*_ for different vales of magnetic field strength B at *γ* = 0.02, *λ* = 0.6.

To understand the effects of magnetic field we plot in Figure 3(a,b) the spin-resolved spectral functions. Figure 3(a) shows the behavior of the down-spin spectral function while Figure 3(b) gives the variation of the spin- up spectral function. It is evident from Figure 3(a) that with increasing *B*, the peaks of the spin-down spectral function rise up and shift towards right in the *ω* – scale. The rise in the side peaks are however only marginal. Figure 3(b) shows that in the spin-up case also, the peaks rise up with increasing magnetic field but shift towards left in the *ω* – scale. In this case however, significant rise in the side peaks is observed. In Figure 4(a,b) we show the variation of the spin resolved spectral functions (*A*_↑_ *and A*_↓_) with respect to the bias voltage *V*_*b*_ for a few values of *B*. In general, the qualitative behavior of *A*_↑_ *and A*_↓_ with *V*_*b*_ are similar. As *V*_*b*_ increases, the spectral function increases, albeit, slowly at small values of *V*_*b*_ and relatively rapidly at large *V*_*b*_. The rapid increase in the spectral function at large *V*_*b*_ is presumably because of the non-Ohmic effect at large bias voltage. We find that in the case of *B* = 0, *A*_↑_ *and A*_↓_ have the same behavior but as *B* is increased, *A*_↑_ *and A*_↓_ behave in an opposite way. While *A*_↑_is found to decrease with *B*, *A*_↓_ increases with B. This is because of the lifting of spin-degeneracy by the magnetic field. As *B* increases, the spin-up level is shifted down and the spin-down level is shifted up.

**Figure 5:**
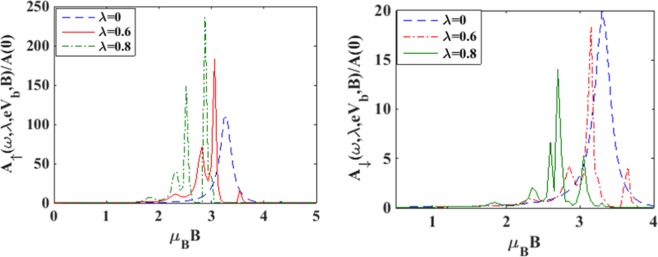
*A*_↑_ *and A*_↑_ *vs λ vs B* for a few values of *λ* at *eV*_*b*_ = 0.5, *γ* = 0.02.

**Figure 6:**
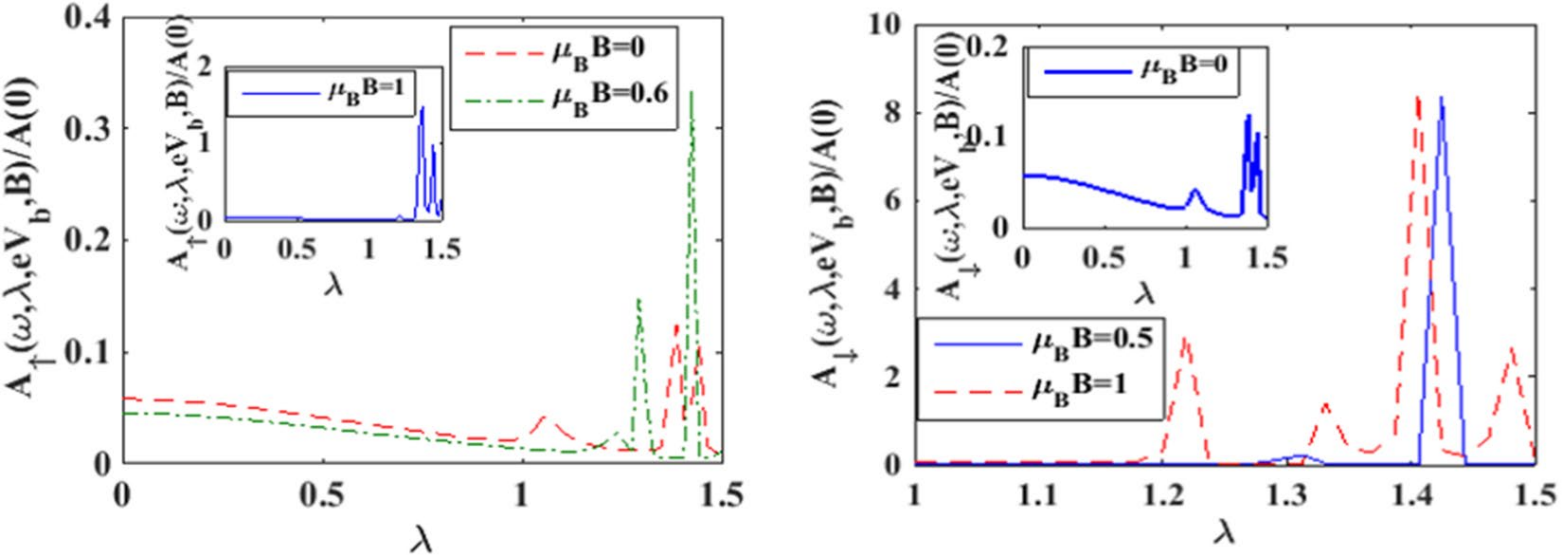
*A*_↑_ and *A*_↓_ *vs λ* for a few values of *μ*_*B*_*B* at *γ* = 0.02 *and eV*_*b*_ = 0.5.

To see the variation of the spectral function with magnetic field (*B*) we plot in Figure 5(a,b), the spin resolved spectral functions with respect to *B* for a few values of *λ*. One can see from Figure 5(a) that in the case of *λ* = 0, *A*_↑_ exhibits a peak at some value of *B*. With increasing *λ*, this peak becomes sharper and shifts towards left (i.e., towards lower values of *B*). Also a few side peaks develop around the main peak. Figure 5(b) shows that *A*_↓_ behaves essentially in a similar way with respect to *B* as *A*_↑_ except that now the main peak-height decreases with increase in *λ*. To unravel the dependence of e-p interaction on the spectral functions directly, we plot *A*_↑_ *and A*_↓_with *λ* for a few values of *B* in Figure 6(a,b). For *B* = 0, the behavior of *A*_↑_ *and A*_↓_ are same but for non-zero values of *B*,the behavior of *A*_↑_*andA*_↓_ are even qualitatively different.

**Figure 7:**
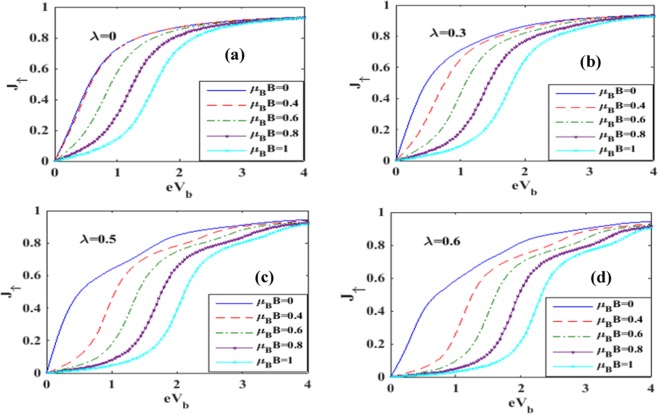
Spin-up current as a function of bias voltage for different values of e-p interaction and magnetic field.

**Figure 8:**
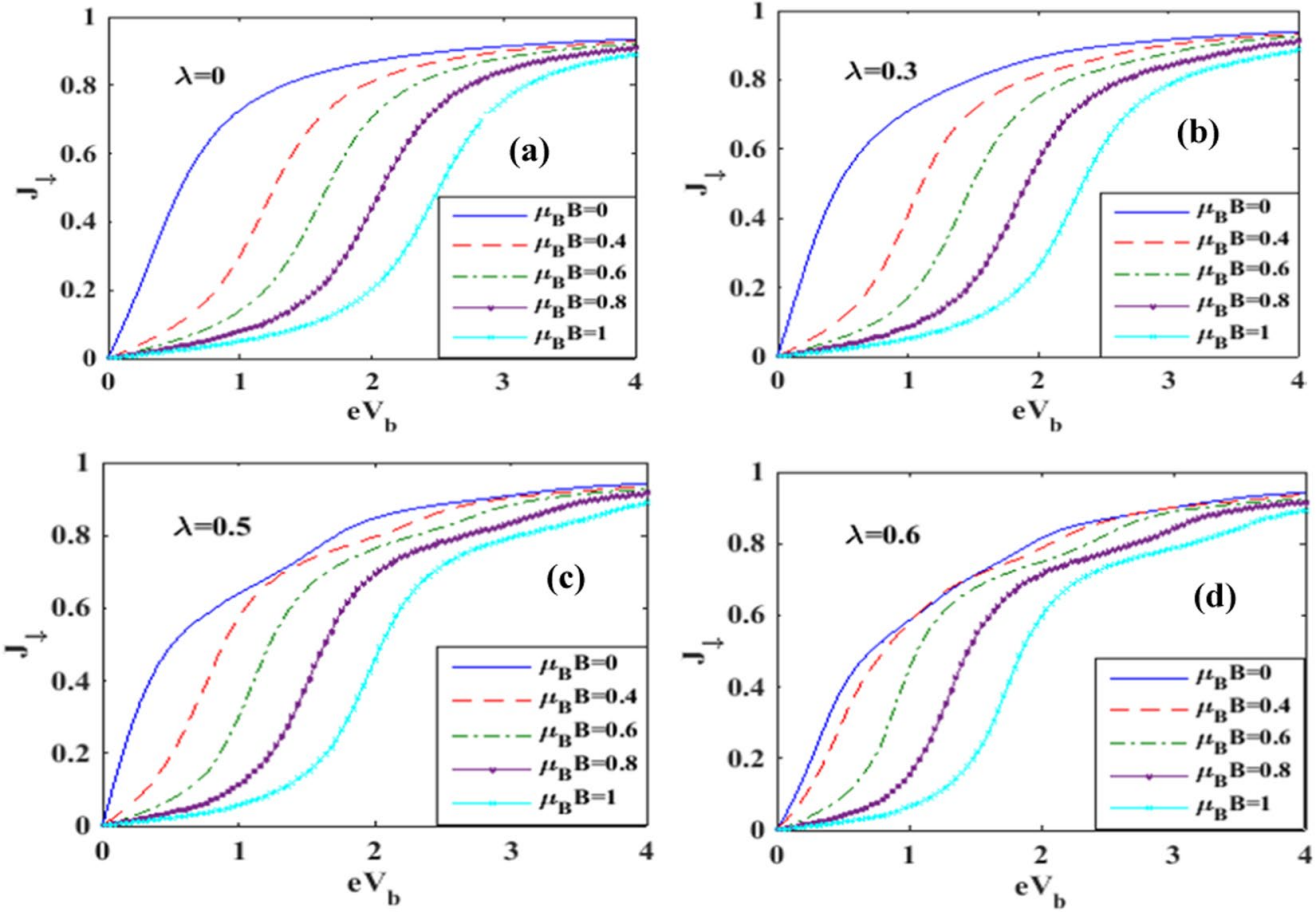
Spin-down current as a function of bias voltage for different values of e-p interaction and magnetic field.

**Figure 9:**
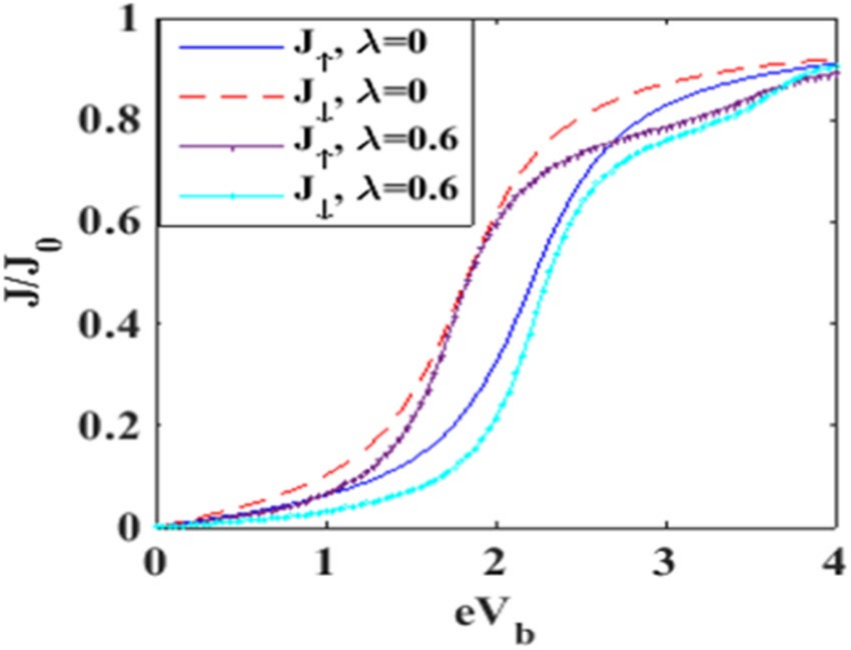
Spin polarized current densities as a function of eV_b_ for different values of *λ* at B = 1.

In Figure 7(a–d) we plot the spin-up tunneling current *J*_↑_ with respect to the bias voltage *V*_*b*_. Figure 7(a) shows the results for a few values of *B* in the case of *λ* = 0. For *B* = 0, the current shows an ohmic behavior at low values of *V*_*b*_ and it appears to reach a saturation value as the voltage is increased. This can be explained in a simple way. As *V*_*b*_ is raised, the Fermi level in the left lead goes up and consequently, more electrons from the source can enter into the QD causing an enhancement in the current. However the quantum dot can accommodate a restricted number of electrons and therefore we expect the current to reach saturation if *V*_*b*_is raised beyond a certain value. In the case of *B* ≠ 0, the two-fold degeneracy of the QD energy level with respect to spin is removed resulting in two energy levels. The spin-up level is shifted down and the spin-down level is shifted up. So the tunneling of the spin-up electrons is precluded unless *V*_*b*_ is high enough to bring down the drain Fermi level to match the spin-up QD level. This causes zero spin-up current to flow in the drain channel. As *V*_*b*_ is further raised, the drain-electron Fermi level comes down below the spin-up level of the QD and then the spin-up tunneling current behaves essentially in an ohmic way and subsequently reaches saturation on the same ground as mentioned in the case of *B* = 0. As *B* is increased, the splitting of the energy level of QD increases. Consequently, *J*_↑_ continues to be zero up to a larger *V*_*b*_ value. In Figure 7(b), we show the behavior for *λ* = 0.3. The current behaves qualitatively in the same way as depicted in Figure 7(a), except that now it is a little lower because of a decrease in the electron mobility due to polaron formation. The reduction in electron mobility due to polaron formation is more pronounced in Figure 7(c,d). Figure 8(a–d) describe the variation of spin-down current densities in the absence and presence of e-p interaction at different values of the magnetic field *B*. Again we see that the magnitude of the current density increases with *V*_*b*_ and eventually saturates. This is of course the behavior that is expected. The appearance of shoulders in Figures 7 and 8 with increasing e-p coupling has been discussed by Khedri *et al*.^33–35^ and Luffe *et al*.^23^. We show a comparison between the spin-up and spin-down current in Figure 9. The figure shows that in the case of *λ* = 0, the magnitude of the spin-up current density is lower than that of the spin-down current density up to a certain bias voltage. The explanation is simple. The spin-up electron levels are lowered by the magnetic field while the spin-down electrons are raised and as a result the current is lower in the case of spin-up electrons because of the smaller tunneling probability of the electrons from the QD to the right lead. In the case of *λ* ≠ 0, the behavior of the current density looks a little more complicated. It turns out that at lower bias voltage, the spin-up current density is higher while at a certain bias voltage, there is a crossing behavior and beyond this bias voltage, the spin-up current density becomes higher. The reason for this strange behavior is not very clear.

**Figure 10:**
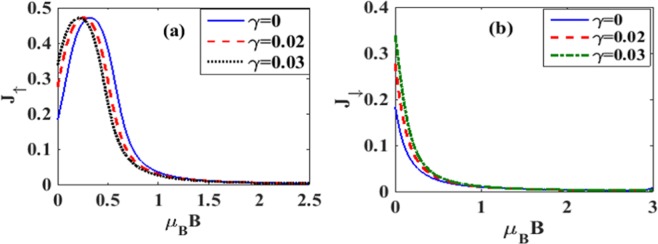
*J*_↑_ and *J*_↓_ vs B with *λ* = 0.6 *and eV*_*b*_ = 0.5 for different values of *γ*.

**Figure 11:**
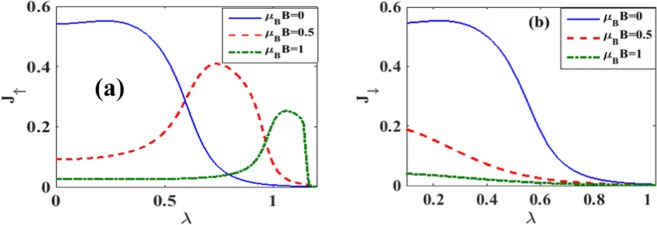
*J*_↑_ and *J*_↓_ vs *λ* for different values of *B* at eV_b_ = 0.5, *γ* = 0.02.

**Figure 12:**
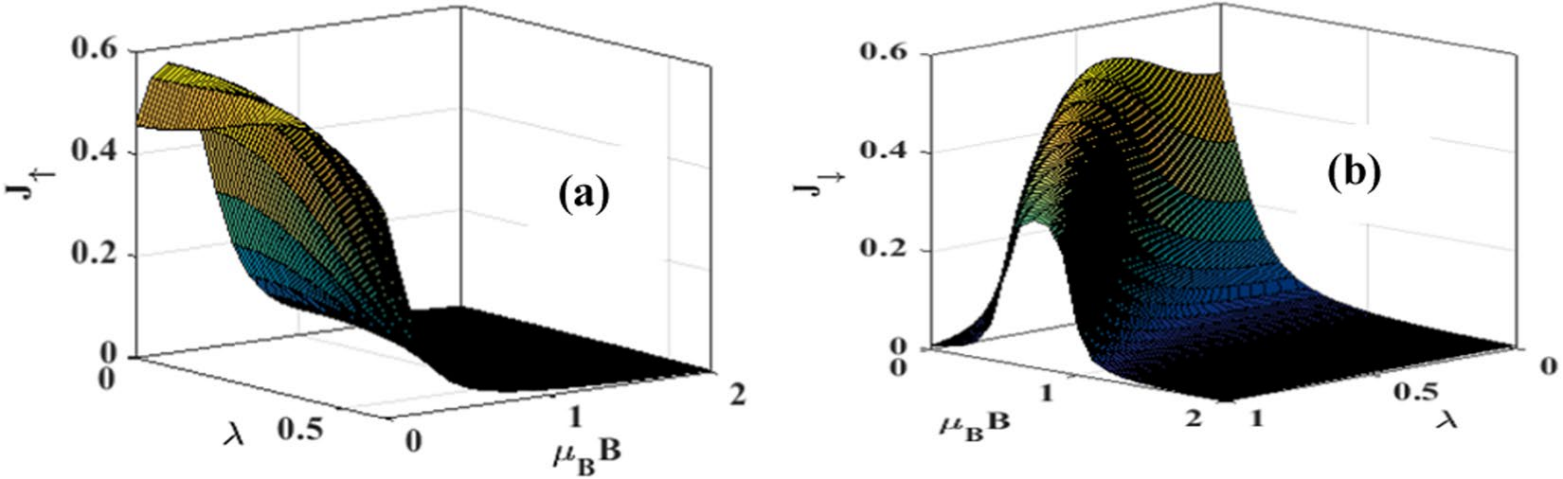
Three dimensional plots of Spin polarized current densities for eV_b_ = 0.5 as a function of both *λ* and B.

In Figure 10(a,b) we directly plot *J*_↑_ and *J*_↓_ vs. *B* for different damping rates with *λ* = 0.6. Figure 10(a) gives results for *J*_↑_ whereas Fig. 10(b) gives results for *J*_↓_. From Figure 10(a) it is clear that the current initially increases with increasing magnetic field, but above a certain magnetic field it decreases and finally becomes zero. The explanation of this behavior is again simple. The magnetic field removes the spin-degeneracy of the QD states and consequently, the spin-up states are shifted down and the spin-down states up. As the magnetic field is increased, spin-up levels are further shifted down and thus allow more electrons to conduct leading to a larger current. But there are two effects that inhibit the current as the magnetic field reaches some critical value. One is the lesser availability of unoccupied states in the QD and the other is the lower probability of tunneling of electrons from the QD to the right lead of SMT. As a result, beyond a certain value of the magnetic field, current starts decreasing and eventually becomes zero. Figure 10(b) shows that the spin-down current decreases monotonically with increasing *B*. As the magnetic field strength is raised, the spin-down levels are shifted up making it more difficult for the electrons to tunnel from the left lead to the QD. This leads to a reduction in the current with increasing magnetic field. Also the dissipation increases the current as expected. Figure 11(a,b) directly show the *λ* – dependence of *J*_↑_ and *J*_↓_ respectively for different values of *B*. Figure 11(a) shows effects on the spin-up electrons of the QD. For a non-zero magnetic field, the spin-up electron levels are shifted down favouring electron tunneling. But there are two effects that inhibit the current if the magnetic field is increased. One is the lesser availability of unoccupied states in the QD and the other is the lower probability of tunneling of electrons from the QD to the right lead of SMT. Polaronic interaction also affects in two ways. One, it reduces the energy which may increase the current and the other is the reduction in mobility due to polaron fomation. Thus there are several competing processes leading to a maximum structure in *J*_↑_. Mathematically, just because of the polaronic effect, the behavior of *J*_↑_ is essentially dominated by the factor: ${\lambda }^{2}{e}^{-{\lambda }^{2}}$. Thus at small *λ*, *J*_↑_ increases with *λ* almost quadratically while at large *λ*, it decreases in a Gaussian way. This leads to a maximum in *J*_↑_ as a function of *λ*. For the spin-down electrons, the energy levels are shifted up by the magnetic field. Thus in this case, *J*_↓_ is expected to decrease with *λ*. Figure 12 show the three-dimensional plot of spin-polarized current densities with respect to *λ* and *B*.

**Figure 13:**
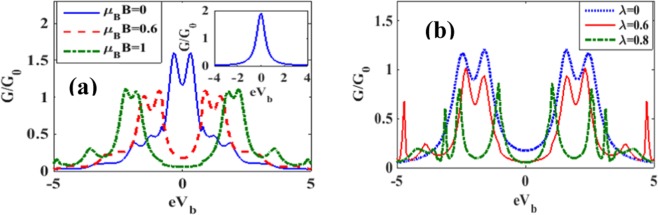
G/G0 vs eV_b_: (**a**) for different values of B with *λ* = 0.6. (Inset: *λ* = *γ* = *B* = 0); (**b**) for different values of *λ* with *μ*_*B*_*B* = 0.5, *γ* = 0.

In Figure 13 we plot the differential conductance (G) with respect to the bias voltage. The inset in Figure 13(a) shows the behavior for *λ* = *γ* = *B* = 0 while the main figure describes the behaviour for *λ* = 0.6 and *γ* = 0.02 for different values of *B*. We observe that in the case of *λ* ≠ 0, even the peak in the *B* = 0 graph undergoes a split. As *B* is increased, the separation between the peaks increases and also each peak splits into two peaks. Also some side peaks appear at a higher bias voltage because of e-p interaction. Each peak, as already pointed out, suggests the possibility of an excitation. Thus the number of energy levels available for participating in transport in the *V*_*b*_ – window increases with *B*. Figure 13(b) shows the variation of *G* with *V*_*b*_ for different *λ* values in the absence of damping. As expected, *G* reduces with *λ*.

**Figure 14:**
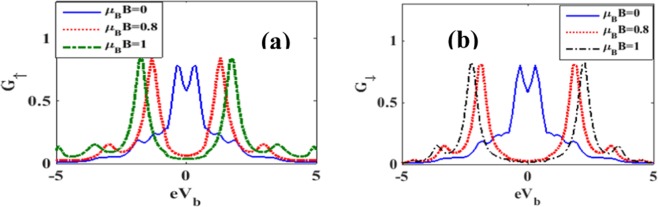
*G*_↑_ and *G*_↓_ vs eV_b_ for different values of B at *λ* = 0.6, *γ* = 0.02.

**Figure 15:**
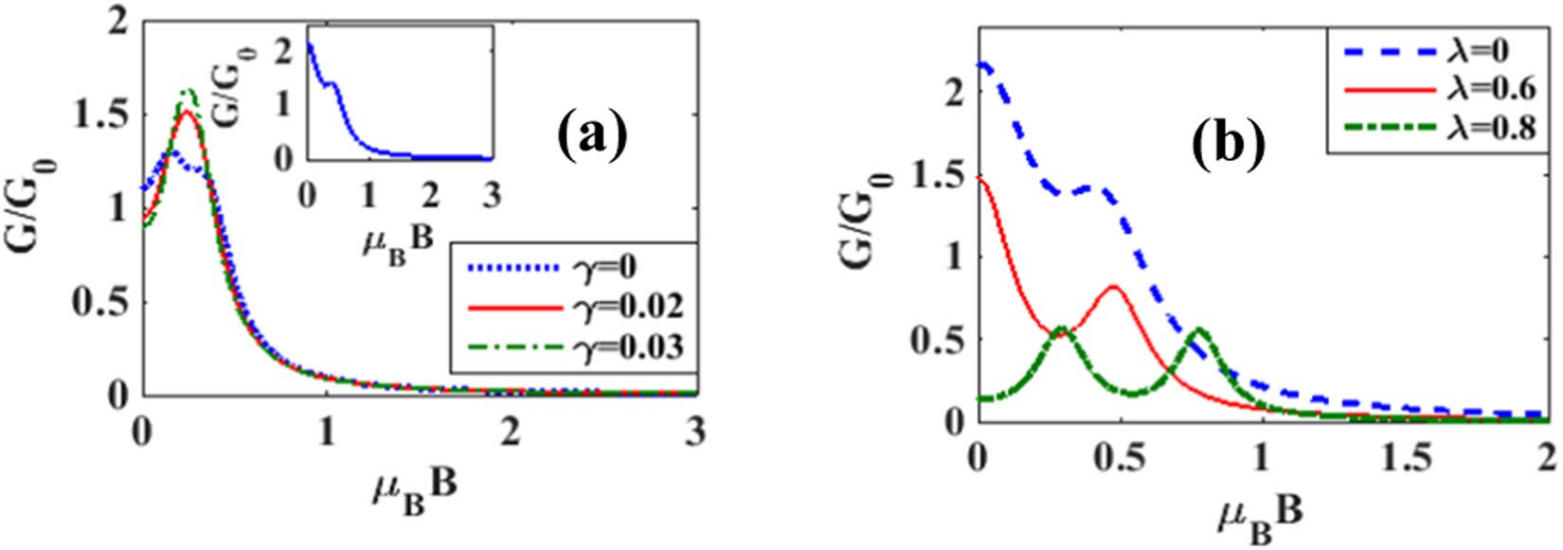
*G*/*G*_0_ vs *B*: (**a**) for a few values of *γ* with *λ* = 0.5 *and eV*_*b*_ = 0.5 (inset *G*/*G*_0_ vs *B for λ* = *γ* = 0 *and eV*_*b*_ = 0.5); (**b**) for a few values of λ with*γ* = 0 *and eV*_*b*_ = 0.5.

**Figure 16:**
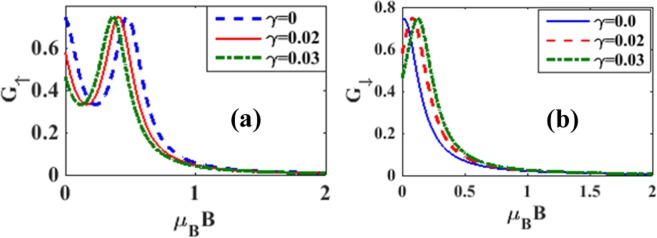
*G*_↑_ and *G*_↓_ vs B for a few values of *γ* at *λ* = 0.6 *and eV*_*b*_ = 0.5.

**Figure 17:**
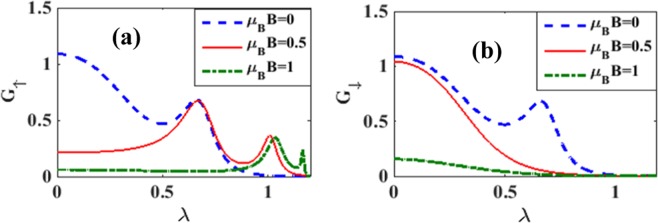
*G*_↑_ and *G*_↓_ vs *λ* for a few values of *B* at *eV*_*b*_ = 0.5, *γ* = 0.02.

The effects of magnetic field on the variation of spin polarized differential conductance *G*_↑_ and *G*_↓_ with *V*_*b*_ are plotted in Figures 14(a,b) for different magnetic fields. To see directly the effect of magnetic field on the differential conductance, we plot in Fig. 15(a), *G* as a function of *B* for *λ* = 0.5 and different values of *γ*. The inset in Figure 15(a) gives results for *λ* = *γ* = 0. The figure shows that in the case of = *γ* = 0, *G*, in general, decreases with increasing *B* which is the expected behavior in view of the localizing effect of the magnetic field. However in a certain window of the magnetic field, *G* shows a small shoulder. In the case of *λ* ≠ 0, *G* in general gets reduced and decreases more rapidly with increasing *B*. Interestingly, however, the shoulder observed in the case of *λ* = 0 develops into a peak in the presence of e-p interaction and this peak height increases with increasing damping rate. Figure 15(b) shows the variation of *G* with respect to *B* for a few values of *λ* in the absence of damping. The shoulder develops into a peak structure as *λ* increases. In fact, for *λ* = 0.8, *G* develops two peaks. In Figures 16(a,b) we plot *G*_↑_ and *G*_↓_ with respect to *B* for a few values of *γ*. The behavior can be explained from the Figures 7 and 8. To study the dependence of *G*_↑_ and *G*_↓_ on e-p interaction, we plot in Figure 17(a,b) *G*_↑_ and *G*_↓_ vs. *λ* for a few values of B. The figures can be explained using Figure 11.

**Figure 18:**
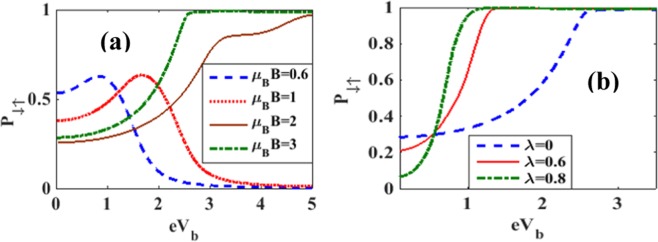
*P*_*σ*, −*σ*_ vs *eV*_*b*_: (**a**) for a few values of *B* with *λ* = 0.5 & *γ* = 0.02; (**b**) for a few values of *λ* with B = 3 & *γ* = 0.02.

**Figure 19:**
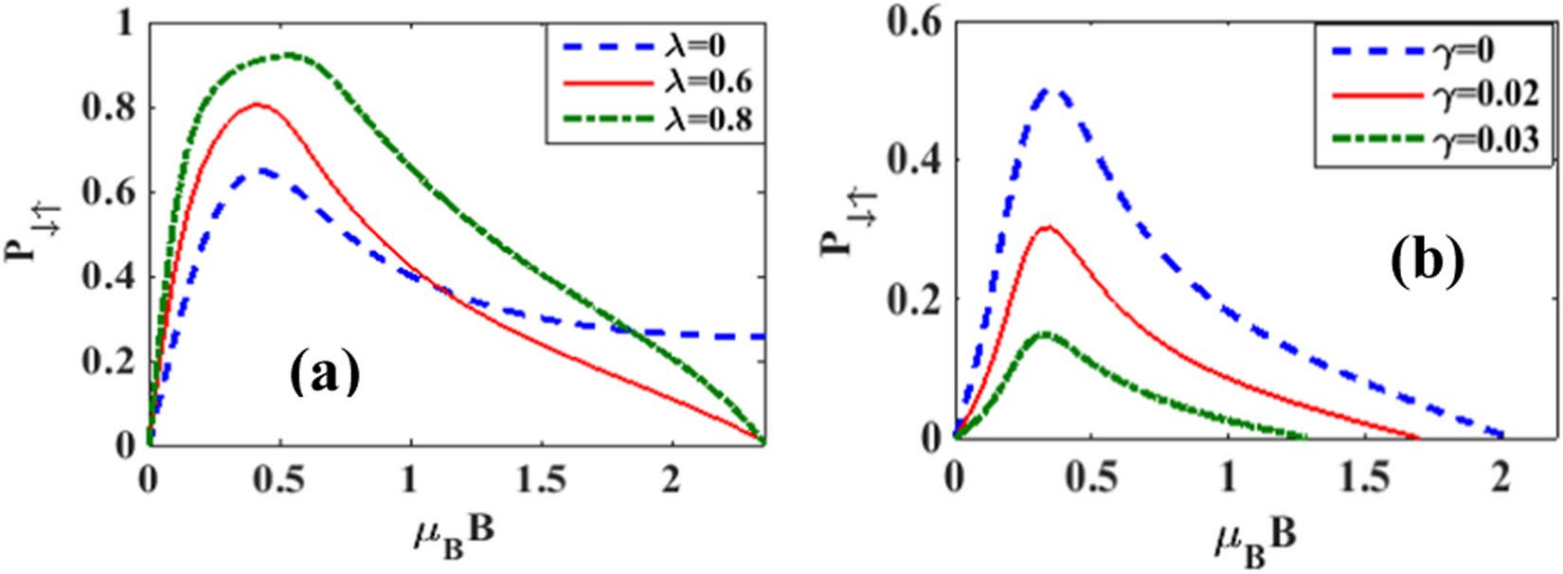
*P*_*σ*, −*σ*_ vs *B*: (**a**) for a few values of *λ* with *γ* = 0 *and eV*_*b*_ = 0.5; (**b**) for a few values of *γ* with *λ* = 0.5 *and eV*_*b*_ = 0.5.

Figure 18(a) shows the behavior of the spin polarization parameter *P*_*σ*, −*σ*_ with respect to *V*_*b*_ for a few values of *B*with *λ* = 0.5 and *γ* = 0.02. At large *B*, *P*_*σ*, −*σ*_ increases with *V*_*b*_ and saturates to a constant as *V*_*b*_ reaches a critical value which is dependent on *B*. At lower magnetic fields however, *P*_*σ*, −*σ*_ initially increases with *V*_*b*_, attains a maximum and then reduces monotonically to zero. Figure 18(b) shows that at low *V*_*b*_, *P*_*σ*, −*σ*_ decreases with increasing *λ* and above a critical value of *V*_*b*_, *P* increases with *λ*. Figure 19(a) shows that in the case of *λ* = 0 = *γ*, *P*_*σ*, −*σ*_ initially increases with *B*, attains a maximum and then reduces with further increase in *B* and eventually reaches a finite saturation value. In the presence of e-p interaction, the initial behavior of *P*_*σ*, −*σ*_ is same but finally *P*_*σ*, −*σ*_ goes to zero as *B* becomes large. Figure 19(b) shows the behavior of *P*_*σ*, −*σ*_ with *B* for different values of *γ*. The behavior is qualitatively similar to that observed in the case of *P*_*σ*, −*σ*_ vs. *B* for non-zero *λ*. Furthermore, *P*_*σ*, −*σ*_ is seen to decrease with increasing dissipation.

**Figure 20:**
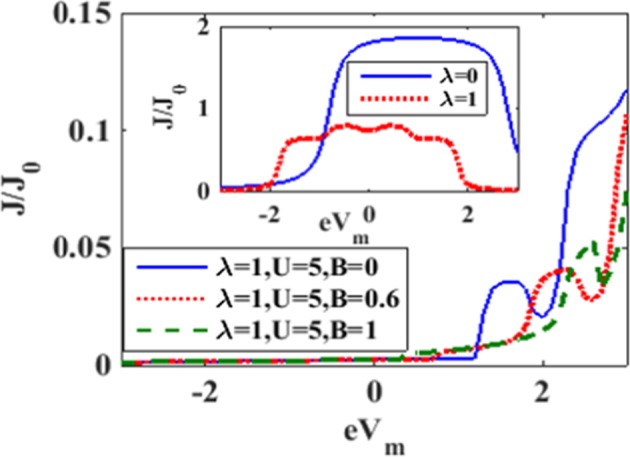
*J*/*J*_0_ vs *eV*_*m*_ at *U* = 5, *eV*_*b*_ = 3.6 for a few values of *B*. (Inset: *J*/*J*_0_ vs *eV*_*m*_ at *U* = 0, *eV*_*b*_ = 3.6 and *B* = 0).

Figure 20 shows the variation of J/J_0_ with respect to mid-voltage eVm for a few values of B with *λ* = 1 and *U* = 5. In the inset we have presented the results of J/J_0_ for *U* = 0, *B* = 0, *eV*_*b*_ = 3.6 and λ = 0 and 1 as obtained by Chen *et al*.^25^. One can observe that both e-p interaction and the magnetic field reduce the current density. Furthermore, the peaks get shifted to the positive mid-voltage side in the presence of e-e interaction and the magnetic field.

**Figure 21:**
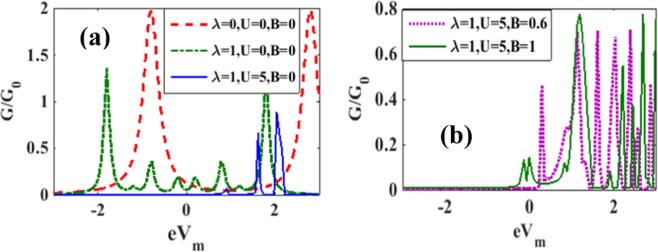
*G*/*G*_0_ vs *eV*_*m*_: (**a**) for *B* = 0, *eV*_*b*_ = 3.66, *U* = 0 *and* 5, *λ* = 0 *and* 1; (**b**) for different values of *B* at *U* = 5, *eV*_*b*_ = 3.6.

In Figure 21(a) we plot *G*/*G*_0_ vs. eV_m_ in the absence of the magnetic field with *λ* = 1, and *U* = 5^25^. We also show the results for *U* = 0, and λ = 0and1 as obtained by Chen *et al*.^25^ to see the effect of e-e interaction. In the case of *λ* = 0, *G* has two peaks asymmetrically placed around *eV*_*m*_ = 0. At λ = 1, the peaks become shorter and sharper and become symmetric with *eV*_*m*_ = 0. Also a few symmetric side peaks appear. As the onsite correlation is increased to U = 5, the peaks become shorter and shift to the right side of *eV*_*m*_ = 0 and G becomes zero for lower positive values of eV_m_ and the entire range of the negative mid-voltage. In Figure 21(b) we display the behavior of *G*/*G*_0_ with respect to the mid-voltage eV_m_ for λ = 1, *U* = 5 with two values of *B*. For *B* = 0.6 we observe a large number of peaks. As *B* is increased to 1, peak structures change.

**Figure 22:**
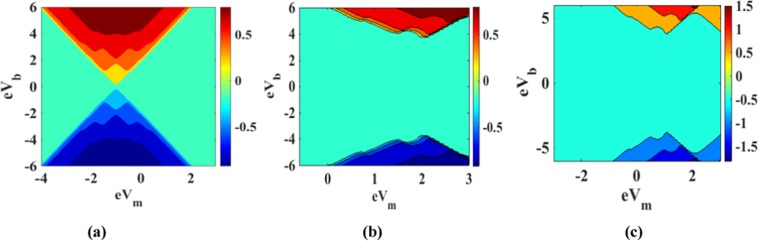
Map of *J* in the V_b_ − V_m_-space: (**a**) for *λ* = 1, *B* = 1 and *U* = 0; (**b**) for *λ* = 1, *B* = 0 and *U* = 5; (**c**) for *λ* = 1, *B* = 1 and *U* = 5.

Figure 22(a–c) give the map of *J* in the (V_b_ − V_m_) - plane for three sets of parameters *λ* = 1, *B* = 1, *U* = 0; *λ* = 1, *B* = 0, *U* = 5 and *λ* = 1, *B* = 1, *U* = 5 respectively. Comparison of Figure 22(a) with the corresponding figure of ref.^25^ suggests that the magnetic field shifts the current density towards the left on the mid-voltage axis leading to an asymmetry in the current density with respect to the zero of the mid-voltage. One can also observe several plateaus corresponding to different values of the current density. Figure 22(b) shows that the current density is reduced in the presence of e-e interaction. Also the current density has a non-zero value only on the positive side of the mid-voltage. Figure 22(c) shows the plot of the current density map in the presence of both e-e interaction and the magnetic field. As expected, the heights of the plateaus are reduced and also current map shift towards the right side in the mid-voltage axis.

**Figure 23:**
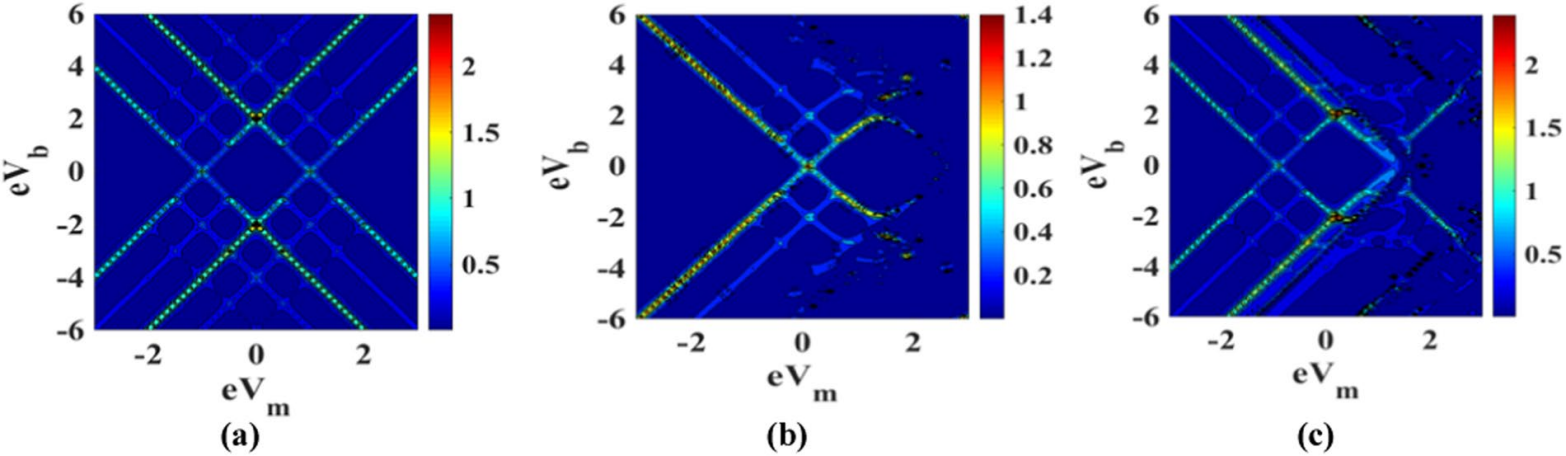
Map of G in V_b_ − V_m_-space: (**a**) for *λ* = 1, *B* = 1 and *U* = 0; (**b**) for *λ* = 1, *B* = 0 and *U* = 5; (**c**) for *λ* = 1, *B* = 1 and *U* = 5.

Figure 23(a–c) present the map of the differential conductance G in the (V_b_ − V_m_)-plane for three sets of parameters *λ* = 1, *B* = 1, *U* = 0; *λ* = 1, *B* = 0, *U* = 5 and *λ* = 1, *B* = 1, *U* = 5. Comparison of Figure 23(a) with the corresponding map given in ref.^25^ shows that magnetic field splits each of the peaks in the *G*-map into two. Figure 23(b) shows the role of the e-e interaction on the *G*-map. It is evident that the e-e interaction reduces the differential conductance. One can also observe in this map some chaotic behavior on the positive side of the mid-voltage. The reason of this random behavior is however not clear. Figure 23(c) presents the effect of both e-e interaction and the magnetic field besides the e-p interaction. The figure is just the combination of Figure 22(a,b) which is of course the expected behavior.

## Conclusion

We have studied the non-equilibrium transport properties of an SMT device in the presence of el-el and el-ph interactions, external magnetic field and phononic dissipation. We have modeled the device by Anderson-Holstein-Caldeira-Leggett Hamiltonian and used Keldysh Green’s function method to calculate the spectral function *A*, current density *J*, differential conductance *G* and spin polarization parameter *P*_*σ*, −*σ*_. The magnetic field removes the spin degeneracy leading to the splitting of QD energy levels and the peaks of the spectral functions. The magnetic field shifts *A*_↓_ towards the positive energy side and *A*_↑_ towards the negative energy side. As expected, the e-p interaction as well as the magnetic field reduces *J*_↓_ and *J*_↑_ is found to increase with increasing magnetic field up to a certain *B* and then falls to zero. Similar behavior is observed for *J*_↑_ with respect to *λ*. The G-plots also show splitting of peaks due to *B* suggesting the existence of more energy levels available due to the splitting of spin degeneracy. *G*_↑_ and *G*_↓_ are found to reduce with increasing *B* but show an opposite effect with respect to *λ*. In the case of *G*_↑_, peaks develop with increasing e-p interacion while in the case of *G*_↓_, e-p interaction supresses peaks. At a low magnetic field, *P*_*σ*, −*σ*_, as a function of V_b_, initially increses but beyond a certain value of V_b_ it starts decreasing and eventually reduces to zero. At higher magnetic fields, *P*_*σ*, −*σ*_ increases with *V*_*b*_ and finally reaches saturation. As a function of the magnetic field, *P*_*σ*, −*σ*_ decreases with increasing *B* and eventually becomes zero. We have shown that damping rate increases spin polarised current densities, differential conductance and spin polarization parameter. We have also shown that as a function of *V*_m_, *J* and *G* undergo a shift for different values of *B* in the presence of *λ* and *U*. *J* is found to shift towards the positive side of V_m_ as *B* increases. In case of G as a function of V_m_, the number of peaks is found to increase with *B*. The results of the present work suggest that an SMT device with a central QD with Coulomb correlation and e-p interaction can be used as a spin filter in the presence of an external magnetic field.
